# Intervention Strategies for Promoting Occupational Participation in Early Mental Health Occupational Therapy in Japan: Focus Group Discussions With Occupational Therapists

**DOI:** 10.7759/cureus.85600

**Published:** 2025-06-09

**Authors:** Takeshi Sasaki, Atsuko Tanimura

**Affiliations:** 1 Department of Occupational Therapy, Ibaraki Prefectural University of Health Sciences, Ami, JPN; 2 Department of Occupational Therapy, Graduate School of Human Health Science, Tokyo Metropolitan University, Tokyo, JPN

**Keywords:** focus group discussions, mental health services, occupational participation, occupational therapy, reflexive thematic analysis

## Abstract

This study aimed to identify occupational therapy intervention strategies, specific to Japan, for promoting occupational participation in the early stage of psychiatric hospitalization. Online focus group discussions were conducted with 13 occupational therapists with clinical experience in Japanese psychiatric wards. The transcripts were analyzed using reflexive thematic analysis.

Four themes emerged as strategies that Japanese occupational therapists use (or consider necessary) to foster occupational participation in early psychiatric inpatient care: (1) "Identifying client occupations"; (2) "Enhancing motivation for occupational participation", primarily through educational interventions; (3) "Providing opportunities for occupational experiences that facilitate change"; and (4) "Addressing difficulties in occupational participation arising from mental disorders". Although therapists must contend with the distinctive challenges of an early stage of hospitalization, these strategies represent ongoing iterative efforts to support clients.

The findings underscore the need to deepen clients’ understanding of occupations, boost motivation through early educational engagement, elicit change through actual occupational experiences, and mitigate barriers caused by mental disorders so that meaningful occupational participation can take place. Taken together, these results suggest that occupation-focused interventions can leverage occupational therapists’ expertise and contribute to recovery-oriented practices in Japan, even within the limited timeframe of early hospitalization.

## Introduction

In recent years, Japan has advanced community-oriented mental‑health policies. To facilitate this shift, the country has reorganized and expanded acute‑care services while actively shortening psychiatric hospital stays [1]. In 2015, the average length of stay in psychiatric wards in Japan was 275 days, with 87% of newly admitted patients discharged within one year. However, the phenomenon of “revolving door” admissions remains prevalent, with approximately 37% of patients readmitted within one year of discharge [1]. These trends highlight the need for early transition to the community, as well as for interventions to prevent readmission and support community living. There is a global trend toward shorter hospitalizations, with the average length of stay in the Organisation for Economic Co-operation and Development (OECD) countries ranging from 10 to 40 days [2]. This is accompanied by a push to enhance community-based services [3].

Furthermore, the World Health Organization (WHO) emphasizes the need to adopt recovery-oriented approaches to mental health service delivery [3]. There are considered to be two recovery categories. The first, clinical recovery, refers to symptom reduction and functional improvement. Personal recovery, the second category, focuses on building a meaningful and optimistic life despite ongoing challenges related to living with mental disorders [4]. A meta-analysis examining the relationship between clinical and personal recovery among individuals with schizophrenia identified a small-to-moderate correlation between symptom severity and personal recovery. This finding underscores the importance of explicitly addressing personal recovery and including it as a core outcome in mental health research [5]. Participation in meaningful occupations, such as work and leisure, has been shown to contribute positively to personal recovery [6]. Consequently, occupational therapists are increasingly called upon to deliver occupation-focused interventions to support personal recovery. Amid the rapid transformation of mental health systems and service concepts, it is essential for service providers to center their practice on personal recovery while simultaneously facilitating community reintegration and preventing unnecessary readmission. Occupational therapists may play a pivotal role in achieving these goals through occupation-focused practices.

In our previous research, we conducted a scoping review of Japanese literature examining the current state of early occupational therapy, considering the challenges from the acute phase to recovery within one year of hospitalization for people with mental disorders [7]. This review identified a range of early occupational therapy interventions, including exercise programs, activity-based therapies, social skills training, psychoeducation, and training in activities of daily living. These interventions primarily aimed to improve cognitive and psychological functions, interpersonal skills, health management, and social adaptation. Most interventions with clearly defined goals were delivered individually and outcome measures predominantly focused on psychiatric symptoms and interpersonal interactions. However, group programs did not tend to have clearly articulated goals. Furthermore, there was limited evidence regarding interventions targeting occupational participation or personal recovery.

Given this lack of evidence, alongside the increasing emphasis on recovery-oriented practice and the need for community reintegration, there is a clear need to clarify effective strategies for supporting occupational participation from the early stage of psychiatric hospitalization. In response to this gap, this study aimed to explore and organize occupational therapists’ perspectives on early intervention strategies that promote occupational participation. By identifying practice-based strategies grounded in therapists' experiences, the study seeks to contribute to the development of a structured, occupation-focused early occupational therapy program that supports occupational participation and personal recovery in mental health care. To achieve these aims, the authors conducted a series of online focus group discussions (FGDs) with occupational therapists. In this study, “early stage of hospitalization” was operationally defined as the first year following admission.

## Materials and methods

Study design

This study adopted a qualitative research design using FGDs following a semi-structured interview approach. The FGDs facilitated the exploratory collection of diverse professional insights and experiential knowledge to clarify the structure of early psychiatric occupational therapy practices and strategies for promoting occupational engagement. To support infection prevention during the COVID-19 pandemic and to improve accessibility, all FGDs were conducted online. The Zoom videoconferencing platform was utilized, which meets the criteria for web-based FGDs as outlined by Tuttas [8].

Participants

The participants in this study were occupational therapists (OTs). The inclusion criteria were as follows: (1) OTs with experience in providing occupational therapy to individuals with psychiatric disorders in the early stage of inpatient care; (2) OTs actively engaged in scholarly activities (e.g., academic publications and conference presentations) related to occupational therapy in the mental health domain; and (3) OTs who possessed the necessary devices and Internet access to participate in online interviews.

Participants were recruited using a combination of purposive and snowball sampling strategies. As a starting point, the researchers identified and contacted OTs who had published relevant articles in Japan’s occupational-therapy-related academic journals within the past five years to request their participation. These individuals were asked to refer the researchers to other eligible OTs who met the inclusion criteria. Participants received email invitations that included explanations of the study’s purpose, methods, and ethical considerations and provided informed consent before enrollment.

Data collection

Data were collected through online FGDs conducted between February and March 2021. The first author was the primary facilitator and the second author supported them as a co-facilitator. Before each session, the study’s purpose and procedures were reiterated to the participants. Thirteen participants were divided into two groups, with each group participating in two FGDs for approximately 90-120 min.

A semi-structured interview guide consisting of five key questions was developed to explore the structure of early occupational therapy practice in psychiatric settings and the strategies employed to promote clients’ occupational participation (Table 1).

**Table 1:** Interview Guide

Questions
1. Please describe the content of your occupational therapy practices in the early stage of psychiatric hospitalization.
2. What types of interventions are you implementing to prevent rehospitalization and support continued community living?
3. What practices do you use to promote occupational participation among psychiatric inpatients?
4. What types of assessments or interventions do you consider necessary to promote occupational participation?
5. Are there any additional points you believe are important regarding early inpatient occupational therapy?

The FGDs were concluded when all participants had answered the questions and indicated that they had no further contributions. When no new information emerged, data saturation was considered to be achieved [9].

Data analysis

Data were analyzed using reflexive thematic analysis (RTA), which is a methodology for identifying ideas within qualitative data and organizing them into themes based on central concepts [10]. The six phases of RTA (familiarization with the dataset; coding; generating initial themes; developing and reviewing themes; refining and naming themes; and writing up) were applied.

Phase 1: Familiarization With the Dataset

The first author began the analysis by reading the transcripts repeatedly to become familiar with the content. During this phase, notes were made on key aspects that stood out. This process facilitated a deeper understanding of participants’ perspectives and helped build a foundation for coding. Member checking was conducted with the participants to confirm the accuracy of the transcribed data.

Phase 2: Coding

Transcripts were carefully read, and with a focus on the aim of the study, relevant segments were manually coded using an inductive approach. The first author highlighted and assigned concise semantic codes that captured the meaning of each segment.

Phase 3: Generating Initial Themes and Subthemes

In phase 3, codes that shared conceptual similarity were grouped together to form initial themes and subthemes. This phase focused on searching for themes and involved interpretive work to identify patterns of meaning in relation to the research question.

Phase 4: Developing and Reviewing Themes

Each preliminary theme and subtheme was reviewed against the original dataset to assess coherence and relevance. During this process, some themes were refined, merged, or restructured. The thematic structure was further developed through analytic discussions involving not only the first and second authors but also the wider research team, ensuring conceptual clarity and grounding in the data.

Phase 5: Refining and Naming Themes

Themes were refined and clearly named to capture their central concepts. Names were selected to concisely and informatively convey the core ideas of each theme. The structure was reviewed again to ensure internal consistency and distinctiveness between themes. Ideas deriving from discussions with co-authors were also revisited and critically evaluated.

Phase 6: Writing up

In the final phase, the themes were presented alongside illustrative quotes from the data. Interpretive commentary was provided to contextualize each theme in relation to the research aim and relevant literature. Throughout the process, the first author maintained a reflexive stance, and ongoing discussions with the second author and research team supported the clarity, transparency, and credibility of the analysis.

Reflexivity and trustworthiness

This study followed Braun and Clarke’s RTA framework, which encourages actively using researchers’ subjectivity and positionality as analytical resources. The first author is an external occupational therapist with no prior relationship with the participants and clinical experience in psychiatric inpatient units and daycare settings. At the time of this study, the first author worked as a university faculty member in education and research. Recognizing that our clinical interest and experience in psychiatric occupational therapy could influence interpretations of participants’ narratives, we maintained a reflexive stance throughout the analysis. Taking a constructionist approach, we aimed to understand participants’ narratives to derive implications for occupational therapy interventions during the early stages of psychiatric hospitalization. Ongoing self-reflection and discussions within the research team enhanced the transparency and credibility of the interpretation process.

## Results

Thirteen occupational therapists (two women and 11 men) participated in this study. The mean age was 38.5 years (SD=6.68). The mean number of years of occupational therapy experience was 11.6 years (SD=5.17), ranging from a minimum of six years to a maximum of 24 years. The total duration of the FGDs was 480.4 min, with a duration of 252.33 min for Group 1 and 228.07 min for Group 2.

This study examined the perspectives of occupational therapists on the structure of early mental health occupational therapy practice and intervention strategies to promote occupational participation. However, this research focuses on the latter component. The RTA identified four overarching themes, each containing multiple subthemes that describe specific practices employed by occupational therapists (Table 2).

**Table 2:** Reflexive Thematic Analysis of Intervention Strategies for Promoting Occupational Participation in Early Mental Health Occupational Therapy

Themes	Subthemes	Occupational therapists' perspectives
Identifying client occupations	Utilizing interviews and occupational therapy assessment tools	Interviews and assessment tools were regarded as effective strategies for identifying client occupations.
	Identifying occupations based on client’s interests	Identifying occupations based on clients’ interests was seen as effective, and tools such as interest checklists were considered useful for facilitating dialogue and eliciting narratives about meaningful occupations.
	Combining observation and information-gathering methods	Observation was considered an important strategy for identifying occupations, particularly when clients had difficulty communicating verbally or articulating their interests.
	Identifying occupations through peer interactions	Peer interactions in informal or group settings were seen as valuable for uncovering clients’ interests and intentions that might not surface during individual sessions or formal interviews.
	Making thoughtful efforts to obtain information on occupations	Gathering information about meaningful occupations required flexible engagement based on clients’ early hospitalization conditions.
	Considering the client’s life history	Focusing on clients' lives before hospitalization and their expected roles after discharge was considered an effective way of identifying meaningful occupations, especially when insight into illness was limited during early hospitalization.
	Identifying occupations in the early stage of hospitalization	Identifying occupations that clients can healthily and meaningfully engage in was considered particularly important during the early stage of hospitalization to guide recovery and post-discharge support.
Enhancing motivation for occupational participation	Promoting early understanding of occupations through educational interventions	It was considered important to provide education about the meaning and value of occupation from the early stages of hospitalization, in order to enhance clients’ understanding and establish a foundation for collaborative engagement.
	Supporting clients in exploring their occupations	Supporting clients in exploring occupations that are personally meaningful was seen as important for enhancing their motivation for occupational participation.
	Supporting clients in clarifying their post-discharge lifestyle vision	It was considered important to support clients in clarifying their envisioned post-discharge life in order to guide the identification of meaningful occupations and enhance motivation for occupational participation.
Providing opportunities for occupational experiences that facilitate change	Supporting positive change through occupational engagement	Engagement in meaningful occupations was seen as a catalyst for positive change and wider recovery in daily life.
	Supporting the initiation and continuation of new occupational experiences	Supporting clients in exploring and continuing new occupations through experiences offered in occupational therapy was seen as important for promoting sustained occupational participation.
	Supporting connections with others through occupations	Facilitating connection and emotional warmth through occupations was regarded as important in acute psychiatric settings.
Addressing difficulties in occupational participation arising from mental disorders	Assessing factors that hinder occupational participation	Supporting occupational participation was seen as requiring the assessment of both client-related factors and occupational demands, using appropriate tools and life-history information to clarify barriers.
	Promoting health in preparation for occupational participation	Stabilizing symptoms through health-maintaining occupations was seen as foundational, while recovery-oriented occupations were also considered essential for supporting meaningful participation based on clients’ individual needs.
	Structuring the environment to promote occupational participation	Preparing physical and social environments that support clients’ continued participation in meaningful occupations was seen as important for sustaining occupational participation after discharge.

The following subsections present these themes and subthemes, accompanied by direct quotations from participants to exemplify them.

Theme 1: Identifying client occupations

Participants acknowledged that, despite the challenges of the early stage of hospitalization, it remains essential to engage clients through diverse approaches to identify their meaningful occupations and foster occupational participation. They emphasized the importance of identifying clients’ occupations from the outset of their admission. The following seven subthemes illustrate the range of strategies described.

Subtheme 1: Utilizing Interviews and Occupational Therapy Assessment Tools

Participants highlighted the necessity of interviews and dialogues to identify client occupations. Although the value of such engagement was recognized even in the early hospitalization phase, they acknowledged that it was not always adequately implemented. One participant reflected, “I always felt it would be ideal to talk with the client one-on-one about their situation, feelings, and what they want to do. That way, I believe we could have uncovered meaningful occupations and those currently desired.”

In particular, for clients experiencing cognitive decline during the early stage of hospitalization, using occupational therapy assessment tools, such as the Assessment by the Picture Cards for the Elderly with Dementia (APCD) or the Aid for Decision-making in Occupation Choice (ADOC), was considered effective in supporting the occupational therapist's understanding. One participant noted, “We have ADOC here, and I find the illustrations to be a really useful tool, especially for introducing occupations early to clients with lower cognitive function.”

Subtheme 2: Identifying Occupations Based on the Client’s Interests

Participants viewed identifying occupations based on their clients’ interests as effective. Tools such as interest checklists were described as a way to elicit narratives about meaningful occupations. One participant stated, “For clients who have difficulty with verbal sessions, I often use an interest checklist as an introductory tool and then draw out narratives through engaging in those activities.”

Subtheme 3: Combining Observation and Information-gathering Methods

When verbal communication was challenging or clients struggled to articulate their interests, participants stressed the importance of observation. Observing clients’ actions and preferences was positioned as crucial for identifying their occupations. One participant noted, “For clients with severe intellectual disabilities or who are quiet or have difficulty writing, I try to observe a few situations and look for what they engage in with the most initiative or for the longest time.”

Subtheme 4: Identifying Occupations Through Peer Interactions

In group settings, natural interactions between clients sometimes revealed interests that might not emerge in formal interviews or individual sessions. One participant explained, “We had some programs that allowed for casual conversation, and in those relaxed, informal settings - not formal interviews - clients sometimes naturally shared their interests.”

When clients had limited expression owing to their medical conditions, interactions with peers occasionally brought out valuable information that would not be shared with the participants and other staff. One participant noted, “Some clients shared things like, ‘You liked doing that, right?’ or ‘Let’s try that after discharge,’ in peer conversations - things they wouldn’t tell the staff. I could gather useful insights by using the group setting.”

Subtheme 5: Making Thoughtful Efforts to Obtain Information on Occupations

Participants noted that, in the early stage of hospitalization, it was sometimes difficult to obtain information about clients’ occupations because of their illness or a lack of rapport. They described flexible strategies aimed at building rapport, such as greeting the client daily and requesting brief written reflections after each session to gather additional information about meaningful occupations. One participant explained, “I wanted to maintain the relationship, so no matter how little response I got, I always made sure to greet them once a day.”

Another noted the following: "We asked participants to fill out a comment sheet at the end of every group session, noting their impressions and satisfaction - either in words or with numbers. By doing so, we hoped to glean even the smallest clues about which activities might be meaningful for them."

Subtheme 6: Considering the Client’s Life History

Particularly in the early stage of hospitalization, when clients might have limited insight into their condition, participants focused on the client’s prehospitalization life to gather occupational information without directly confronting the psychiatric pathology.

One participant described their approach: "For clients with schizophrenia who have little grasp of why they are hospitalized, I set aside questions about their illness and say something like, 'I know life in the ward can be difficult, but what kinds of things did you usually do at home?' By focusing on their pre‑hospital daily life in this way, I pick up conversation topics and gather information."

Understanding the client’s life before hospitalization and the roles they would be expected to assume after discharge were also used to identify occupations. In the early stage of hospitalization and beyond, participants emphasized the importance of understanding the continuity of clients’ occupations across the past, present, and future as well as the client’s life history.

One participant explained their approach: "I focus on what the client is struggling with and ask the primary occupational therapist to collect information on how they lived at home before admission, the roles they held, the roles they are expected to assume after discharge, and the places where they are likely to spend time once they leave the hospital."

Subtheme 7: Identifying Occupations in the Early Stage of Hospitalization

Despite the challenges of focusing on occupations in acute psychiatric settings, participants emphasized the value of incorporating meaningful occupations, even in the early stage of hospitalization. They highlighted the importance of maintaining occupational perspectives for supporting post-discharge life and recovery.

One participant clarified their perspective: "When I consider the need to intervene in the meaningful daily activities and occupations that a person values - even in the acute phase - I feel it isn’t enough to discharge them just because their psychiatric symptoms have subsided. We need to deliberately focus on the meaningful activities and occupations they still can’t carry out and think about how to improve those areas and help them return to them."

Participants stressed the importance of identifying clients’ valued occupations as early as possible. Another participant noted, “It’s really important to clarify what the client can healthily engage in. I feel the key in early intervention is how quickly we can uncover that.”

Theme 2: Enhancing motivation for occupational participation

This theme captures the importance of increasing motivation for occupational participation and the strategies that occupational therapists use to achieve this goal. Participants emphasized the value of educational interventions that deepen clients’ understanding of occupations, support them in exploring and making decisions about their own occupations, and provide motivation by clarifying a concrete vision of life after discharge.

Subtheme 1: Promoting Early Understanding of Occupations Through Educational Interventions

Participants described the importance of conveying the meaning and value of occupations to clients from the early stage of hospitalization to foster awareness and interest in engaging in occupations. One participant noted, “Regarding the term ‘occupational participation,’ I personally feel it has a meaning similar to ‘personal recovery.’ By explaining this connection to clients, I think it becomes easier for many to understand.”

Another participant also highlighted that an educational approach could encourage patients to reflect on their occupations and lay the foundation for collaborative engagement with therapists: "I strongly believe it is important for occupational therapists to provide education about occupations. Of course, the most important occupations are unique to each individual, so they must be discovered by the clients themselves. Educating them about occupation from the beginning helps them reconsider their own occupations and builds a foundation for shared exploration with the therapist."

Subtheme 2: Supporting Clients in Exploring Their Occupations

Participants expressed that supporting clients to discover and select meaningful occupations was crucial for increasing their motivation for occupational participation. They also underscored the need for ongoing engagement with clients, even during brief hospital stays, to facilitate their selection of occupations with a view toward encouraging post‑discharge participation.

One participant described their approach as follows: "Through regular goal setting and interviews, I try to frequently ask ‘What do you want to do?’ and provide opportunities for clients to think for themselves. I believe that in acute settings, what we can offer is to provide these cues for self-reflection, which is something we can do for many hospitalized individuals."

Subtheme 3: Supporting Clients in Clarifying their Post-discharge Lifestyle Vision

Participants emphasized that helping clients envision their lives after discharge, including what occupations they would like to engage in, can enhance their motivation for occupational participation. They recognized the importance of supporting clients in connecting their preferred occupations to real-life contexts.

One participant noted the following: "Programs put together by OTs often don’t match what the client truly wants to engage in, so I think interventions should be grounded in the client’s own volition - that is, in the life they hope to realize or the aspects they wish to change."

Another participant shared the following narrative: "There was a client who really enjoyed watching music shows. In the hospital, they couldn’t watch late-night programs like Countdown TV because of lights-out times. But after discharge, the freedom to watch those shows became a strong motivator that led them to adhere to medication and successfully transition to community life."

Theme 3: Providing opportunities for occupational experiences that facilitate change

This theme emphasized that engaging in occupations can serve as a crucial means of facilitating client change. Participants described strategies for providing meaningful occupational experiences during hospitalization, particularly in the early stage of hospitalization. They highlighted the importance of supporting change through occupational engagement, promoting new experiences and their continuation, and fostering social connections through occupation.

Subtheme 1: Supporting Positive Change Through Occupational Engagement

Participants noted the significance of enabling clients to engage in meaningful occupations even during the early stage of hospitalization. They emphasized that facilitating engagement in meaningful occupations can trigger positive changes such as enhanced activity levels and broader occupational participation.

One participant shared the following narrative: "During hospitalization, I learned that piano and calligraphy were truly important occupations for her. These activities continued after discharge, and as she received praise from others, she gradually began smiling more. It took several years, but eventually she became more active and able to do various things."

Another participant described their perspective as follows: "When we think about occupational participation, we often refer to domains like work, leisure, and ADLs. It’s difficult to improve all of them at once, but I believe occupations have a synergistic effect. So I focus on interventions that target areas which can lead to an upward spiral in health and well-being."

Subtheme 2: Supporting the Initiation and Continuation of New Occupational Experiences

Participants pointed out that exposing clients to a wide range of activities offered in occupational therapy could open the door to new forms of occupational participation.  They also noted that supporting clients so that they can continue their occupations after discharge was vital for sustaining their participation. One participant stated, “There was a client who started painting after observing others, went on to win awards at a regional art exhibition, and continued attending painting classes after discharge. That experience helped them find an occupation that was meaningful.”

Another participant shared the following narrative: "Some clients tried scratch art for the first time during hospitalization and became completely absorbed. They continued doing it in their hospital rooms, and even when one client was readmitted, they brought back impressive artwork. It made me realize how much that occupation resonated with them."

Subtheme 3: Supporting Connections With Others Through Occupations

The participants recognized the unique value of group-based psychiatric occupational therapy in facilitating interpersonal connections through shared activities. They emphasized that these connections are essential for promoting occupational participation and preventing social isolation. One participant stated, “Unless clients connect with others, nothing really starts. I believe that meaningful occupations are created through interactions with others. Even in acute settings, it’s vital to feel warmth and connection. Occupational therapy can help bridge these connections through shared occupations.” Another noted, “I think it’s important for clients to sense a connection with others through activities and to experience others’ warmth. Ultimately, perhaps it comes down to feeling love-realizing, ‘I am valued,’ and wanting to value someone else.”

Theme 4: Addressing difficulties in occupational participation arising from mental disorders

This theme explores how occupational therapists address barriers to occupational participation that stem from mental disorders. The participants emphasized the importance of assessing such barriers, promoting clients’ health in preparation for occupational participation, and structuring the environment to facilitate engagement. Even in the presence of clinical and environmental constraints, appropriate evaluations and adjustments are essential to enable smoother occupational participation after discharge.

Subtheme 1: Assessing Factors That Hinder Occupational Participation

The participants stated that to support clients’ occupational participation, it is essential to evaluate and clarify the factors that hinder it. They emphasized the importance of drawing on occupational therapy assessment tools and life‑history information to appropriately assess challenges related to both clients and occupations. One participant emphasized, “To enable occupation, we need to assess both the client’s condition and the occupation itself - occupation they are attempting to carry out. Without a clear understanding of these two elements, successful implementation is unlikely, so that step is essential.” Another participant described their use of the Classification and Assessment of Occupational Dysfunction (CAOD) to assess factors that hinder occupational participation. They stated, “When a client strongly refuses participation, I sometimes use the CAOD alongside the primary nurse, comparing it with their life history to consider whether there might have been difficulties in their past lifestyle.”

Subtheme 2: Promoting Health in Preparation for Occupational Participation

Supporting clinical recovery through medication adherence and symptom stabilization is essential for achieving occupational participation. Stabilization of symptoms and continued medication served as a foundation, making it easier to maintain healthy occupational participation in daily life after discharge.

One participant shared the following perspective: "If occupational participation means engaging once again in activities that the person wants to do or that are important to them, then, considering the readiness pyramid or that kind of triangular diagram, the most fundamental level is having stable symptoms and being able to take medication properly…"

In the early stage of hospitalization, clinical recovery, such as symptom stabilization and health maintenance, tends to be the main focus; however, there is recognition of the importance of supporting occupational participation and personal recovery. One participant noted, “I think there are occupations for recovery, and they are necessary, but when focusing on the individual, attention tends to shift more toward health maintenance rather than recovery. I think both are important…”

Subtheme 3: Structuring the Environment to Promote Occupational Participation

Participants discussed the need to adapt to both the physical and social environments to support their occupational participation. They believed that by preparing environments in which clients could engage in meaningful occupations after discharge, they could build daily patterns that incorporated those occupations, thereby promoting ongoing occupational participation.

A participant shared the following narrative: "At first, this client seemed unable to take part in any activities, but we learned she had played the piano and practiced calligraphy since childhood. When she tried both in the OT room, she turned out to be very skilled. Because she had no piano at home, a keyboard was arranged for her, and even after discharge she kept practicing-promising to perform whenever the home‑visit nurse came. She would say, ‘The nurse is coming, so I need to practice,’ and continued working on her pieces at home."

## Discussion

Structure of early mental health occupational therapy practice to promote occupational participation

This study conducted FDGs to explore occupational therapists’ perspectives on intervention strategies for early psychiatric occupational therapy in Japan to promote occupational participation. Based on the narratives shared, four key strategies were identified based on therapists’ accounts, which may inform the understanding of early occupational therapy practices: “identifying client occupations” from an early stage in order to develop and continue occupation-focused support even within the limited period of hospitalization, “enhancing motivation for occupational participation” primarily through educational approaches, “providing opportunities for occupational experiences that facilitate change” and “addressing difficulties in occupational participation arising from mental disorders.” These themes reflect therapists’ perceptions of meaningful practices within the limited timeframe of hospitalization and provide insight into how occupational therapy might support clients in early recovery contexts. The following subsections discuss the significance of each theme in relation to the experiences and interpretations of the participants.

Identifying Client Occupations

This strategy emphasizes the importance of using interviews, observations, and occupational therapy assessments to identify occupations and interests that are meaningful to the clients. While occupational therapy practice has recognized the importance of supporting meaningful occupations, interventions often focus on cognitive or health management skills. Interventions that explicitly identify clients’ meaningful occupations are not always implemented [7]. Individuals living with mental disorders often experience difficulties identifying what they would like to do. This is often due to low self-confidence or high anxiety, such as having the feeling that “their desires are chaotic” [11]. Therefore, in the context of mental health, “identifying client occupations” was perceived as particularly meaningful in supporting occupational participation.

Orui et al. [12] demonstrated the relationship between personal recovery and occupational participation, particularly in leisure activities. Similarly, McKeown et al. [13] reported that both clients and practitioners recognized participation in meaningful occupations, including leisure, as central to understanding recovery and health. Conversely, a focus group study of clients discharged from psychiatric hospitals identified difficulties in initiating leisure activities post-discharge [14]. This suggests the possible importance and utility of identifying occupations during the early stage of hospitalization to support community transition.

A meta-synthesis by Hitch et al. [15] on clients’ experiences of occupational engagement reported its strong links to identity, noting that it serves as a coping mechanism and relates to recovery and health. Furthermore, Hitch et al. [15] found that habits and hobbies are perceived as essential for continued recovery. Moreover, “being able to choose one's occupations” and “feeling like oneself” have been identified as key elements of recovery [16]. Accordingly, identifying meaningful occupations during the early stage of hospitalization may be an important intervention strategy that contributes to both occupational participation and personal recovery.

Enhancing Motivation for Occupational Participation

The model of human occupation (MOHO) [17] positions clients’ motivation (volition) as central to occupational engagement. Since motivation for occupational participation can be significantly impaired due to mental disorders, it is vital to reactivate client volition [18]. Lee et al. [19] found that more than half (52.1%) of their participants experienced barriers or restrictions to occupational participation. Furthermore, Lee et al. [19] identified that many participants had significant problems with motivation for their occupation. These findings suggest that there may be a need for interventions that support motivation for occupational engagement.

Recent studies have shown correlations between motivation and social functioning. Extrinsic motivation was found to be positively associated with interpersonal functioning, intrinsic motivation was positively correlated with personal functioning (e.g., leisure and independence), and amotivation was negatively associated with both forms of functioning [20]. Intrinsic motivation has also been identified as a significant mediator between cognitive and psychosocial functions and is considered a promising therapeutic target [21].

Strategies for supporting motivation included: “promoting early understanding of occupations through educational interventions,” “supporting clients in exploring their own occupations,” and “supporting clients in clarifying their post-discharge lifestyle vision.” The MOHO also links interest to motivation, and previous research suggests that past activity experiences foster continued engagement [22]. Furthermore, educational support that enables clients to independently reflect on their occupational choices is critical. Lipskaya-Velikovsky et al. [23] found that occupation-focused educational programs effectively enhanced diversity and satisfaction with occupational participation. Therefore, to promote occupational participation, motivational support may benefit from incorporating educational approaches regarding the relationship between occupation, health, and recovery. This enables clients to actively consider their own occupations. Such approaches go beyond mere knowledge transfer, aligning with the principles of recovery-oriented practices.

Providing Opportunities for Occupational Experiences That Promote Change

Engaging in occupations from the early stage of hospitalization has been discussed as a catalyst for client transformation and participation. This aligns with the MOHO's theoretical foundation, which asserts that factors related to occupation evolve through engagement. Furthermore, it supports the view that occupational engagement can foster self-awareness and change.

Psychiatric occupational therapy is often group-based and characterized by the therapeutic use of meaningful occupations to support mental health and recovery. Leveraging these characteristics and providing clients with opportunities to engage in various occupations may serve as a catalyst for exploring new occupations and expanding their interests. Furthermore, more individual occupational experiences may promote both internal changes and lead to broader occupational participation. According to the MOHO, clients’ internal characteristics, including volition and performance capacity, are understood to change through engagement in occupation [17]. Thus, in early-stage psychiatric occupational therapy, providing clients with opportunities for occupational experience seemed to foster change and support the construction of an autonomous life.

The importance of forming social connections through occupation was also highlighted. Waks et al. [16] emphasized that feeling connected to others, especially peers with similar experiences, supported recovery in subacute care. These connections can be formed naturally through occupational interactions. Group-based interventions have been shown to promote outcomes such as social bonds, achievement, and peer learning [24]. However, in Japan, the implementation and effectiveness of group-based interventions remain underexplored [7]. In this context, the strategy of “providing opportunities for occupational experiences that promote change” offers practical insights into how group engagement fosters both interpersonal connections and individual change.

Addressing Difficulties in Occupational Participation Arising from Mental Disorders

To support occupational identification, motivation, and engagement, it is necessary to address difficulties in occupational participation arising from mental disorders. In particular, during the acute phase or early hospitalization, patients may experience residual symptoms or confusion. Occupational therapists need to establish a foundation that enables clients to engage in occupations by "assessing factors that hinder occupational participation", implementing interventions aimed at "promoting health in preparation for occupational participation, such as managing psychiatric symptoms and daily life", and " structuring the environment to promote occupational participation".

In interventions aimed at "promoting health in preparation for occupational participation", the participants emphasized the importance of focusing on clinical recovery, such as stabilizing symptoms and managing medication. Clinical and personal recovery exhibit low to moderate correlations [5]. In mental healthcare, readmission remains a prevalent issue, necessitating attention to clinical recovery to support clients’ occupational participation and sustained community living. However, achieving clinical recovery does not guarantee personal recovery [25]. While supporting clinical recovery, a recovery-oriented practice that prioritizes personal recovery is essential, and occupation-focused practice was seen as a core perspective in this approach.

The significance of occupation-focused practice in the early stage of hospitalization and the contribution of this study

The four strategies identified in this study appear to correspond to the two core components of occupational adaptation described in the MOHO: occupational identity and occupational competence. These strategies may provide a practical framework for supporting occupational adaptation among clients in acute psychiatric settings. According to MOHO [17], occupational adaptation is defined as “the development of a positive occupational identity, coupled with the experience of occupational competence over time within the context of one’s environment.” Occupational competence refers to “the ability to sustain a pattern of occupational participation that reflects one’s occupational identity.”

The first strategy, "identifying client occupations", may contribute to the reconstruction of occupational identity by enabling individuals to articulate and reconnect with occupations and roles that hold personal meaning. The second strategy, "enhancing motivation for occupational participation", could be viewed as strengthening the volitional foundations of occupational competence by working on clients’ values, interests, and sense of efficacy. The third strategy, "providing opportunities for occupational experiences that promote change", may help foster occupational competence by creating opportunities for clients to experience and reflect on their capabilities through engagement in occupation. The fourth strategy, "addressing difficulties in occupational participation arising from mental disorders", might support both occupational identity and competence by helping clients navigate internal and external barriers related to mental illness. Taken together, these four strategies may be understood as aligning with the conceptual framework of occupational adaptation in MOHO, particularly through their potential to influence the development or preservation of occupational identity and competence. Within the dynamic and often constrained context of acute psychiatric care, these findings suggest that occupation-based interventions may play a meaningful role in supporting clients’ sense of agency and self-understanding.

Previous studies have highlighted the difficulties of recovery-oriented practice, which shares core values with occupational therapy, such as occupation-focused practice, client centeredness, and the achievement of personally meaningful goals during the early stage of hospitalization. For example, acute care environments are strongly dominated by the medical model, which can result in the unconscious suppression of clients and restriction of occupational opportunities [26]. Additionally, recovery-oriented practices may be difficult to implement in psychiatric inpatient wards because mental health professionals may be skeptical about recovery concepts and outcomes for individuals experiencing acute psychiatric episodes [27].

Furthermore, there is little research on occupational participation in acute psychiatric wards in Japan. There is a clear need to generate new evidence on occupation-focused practices to clarify the clinical role of occupational therapy in this context [28]. Hitch and Lhuede [29] identified that conducting occupation-focused practice, exploring clients’ experiences in therapeutic groups, identifying factors that enhance clients’ occupational engagement, and promoting meaningful and proactive occupational engagement in inpatient settings as research priorities in mental health occupational therapy.

The strategies identified in this study for advancing occupation-focused practice from the early stage of hospitalization may provide guidelines for recovery-oriented practice, supporting the central concepts of occupational therapy client-centeredness and the role of occupational therapists as specialists in occupation-focused practices.

Limitations and future directions

This qualitative investigation explored and described strategies for promoting occupational participation in occupational therapy during early psychiatric hospitalization. As this investigation was based on the narratives of individual participant OTs, the findings are dependent on their experiences and may have limited generalizability. Most participants in this study were men, which may have limited the diversity of perspectives. Although gender-based differences were not evident in the analysis, their potential influence cannot be ruled out. Future research should include a more diverse sample to enhance the applicability of the findings. Furthermore, this study did not examine the actual effects of the identified intervention strategies on occupational participation. In future, the effectiveness of these strategies should be clarified through quantitative and interventional studies.

## Conclusions

This study explored potential intervention strategies to promote occupational participation among individuals with mental disorders in the early stage of hospitalization. Qualitative data from FGDs with occupational therapists were analyzed using the RTA framework. The analysis yielded four intervention strategies: (1) identifying client occupations, (2) enhancing motivation for occupational participation, (3) providing opportunities for occupational experiences that facilitate change, and (4) addressing difficulties in occupational participation arising from mental disorders. These findings highlight key considerations that may inform the development of occupation-focused and recovery-oriented practices in early psychiatric occupational therapy. The results offer preliminary insights that could support therapists in shaping occupation-focused interventions within the context of shortened hospital stays in contemporary Japanese psychiatric care.
